# Misaligned hormonal rhythmicity: Mechanisms of origin and their clinical significance

**DOI:** 10.1111/jne.13144

**Published:** 2022-05-06

**Authors:** Eder Zavala

**Affiliations:** ^1^ Centre for Systems Modelling & Quantitative Biomedicine University of Birmingham Edgbaston UK

**Keywords:** chronodisruption, circadian, hormone dynamics, mathematical modelling, rhythm misalignment

## Abstract

Rhythmic hormonal secretion is key for sustaining health. While a central pacemaker in the hypothalamus is the main driver of circadian periodicity, many hormones oscillate with different frequencies and amplitudes. These rhythms carry information about healthy physiological functions, while at the same time they must be able to respond to external cues and maintain their robustness against severe perturbations. Since endocrine disruptions can lead to hormonal misalignment and disease, understanding the clinical significance of these rhythms can help support diagnosis and disease management. While the misalignment of dynamic hormone profiles can be quantitatively analysed though statistical and computational techniques, mathematical modelling can provide fundamental understanding about the mechanisms underpinning endocrine rhythms, particularly around the question of what makes them robust to some perturbations but fragile to others. In this study, I will review the key challenges of understanding hormonal rhythm misalignment from a mathematical perspective, including their causes and clinical significance. By reviewing modelling examples of coupled endocrine axes, I will address the question of how perturbations in one endocrine axis propagate to another, leading to the more complex issue of disentangling the contribution of each endocrine system to a robust dynamic environment.

## 1 WHAT IS HORMONAL MISALIGNMENT?

Many hormones in the body oscillate with different frequencies and amplitudes, creating a dynamic environment that is essential to maintain healthy homeostasis. A central clock in the hypothalamus governs this dynamic environment, but clock mechanisms are present in all peripheral tissues.^1^ A systems‐level coordination of these clocks is necessary to sustain health in the face of perturbations. This involves key physiological processes including hormonal regulation of the stress response, metabolism, and sleep. Although these hormone rhythms are controlled endogenously, external stimuli, particularly the timing of stressors, meals, sleep, and light exposure can profoundly influence them.^2^ In humans, disruptions to these rhythms are strongly associated with increased morbidity and mortality. Comprehensive studies have measured the effects of forced desynchrony in the light–dark cycle and of meal timing on hormonal rhythmicity and other physiological variables.^3^, ^4^ High‐frequency plasma measurements in healthy individuals subject to overnight wakefulness have shown phase shifts and amplitude changes in cortisol and melatonin profiles.^5^, ^6^ The resulting rhythm misalignment is also characterised by systematic increases in postprandial glucose, insulin, and mean arterial pressure, systematic decreases in leptin and sleep efficiency, increased variability of catecholamines, and an inversion of circadian cortisol profiles.^3^

Misalignment of hormonal rhythms is particularly relevant in rotating shift workers who are at a higher risk of cardiometabolic disease in a dose‐dependent fashion (relative risk [RR]: 1.23 of coronary heart disease, RR: 1.57–1.77 of metabolic syndrome, RR: 1.09–1.40 of type 2 diabetes),^3^, ^7^, ^8^, ^9^ as well as of altered immune function, stroke and cancer due to their imposed behavioural schedule.^10^, ^11^ Up to 15% (20%–30% worldwide) of the UK workforce performs some type of shift work, with up to a third employed in health and social work sectors.^12^ While the detrimental effects of shift work have been the subject of much research, understanding the misalignment of hormonal rhythms in shift workers has been hindered by the lack of suitable measurements in occupational settings. Recent technological advances are making possible to address this challenge. There is now a novel ambulatory microdialysis device, worn on the waist, that enables high‐frequency hormone measurements without the need for blood.^13^ There are also devices that can measure glucose continuously in the form of a shoulder patch and a finger ring that can record physical activity, heart rate variability, body temperature, and sleep structure. Altogether, these technological developments are enabling a high‐resolution characterisation of hormonal rhythms and their external manifestations in real‐life settings.^14^ However, a theoretical framework to understand how rhythm misalignment emerges as a consequence of external disruptors is still lacking.^15^

**FIGURE 1:**
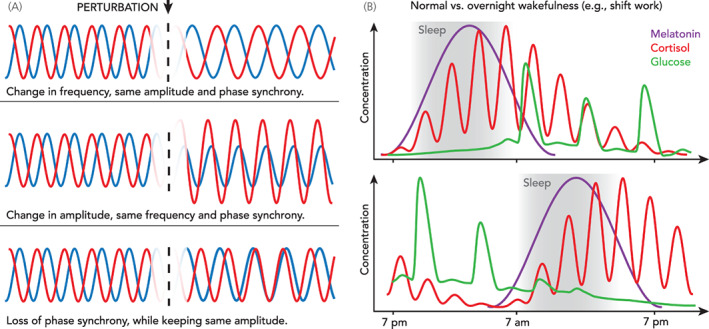
(A) Rhythm misalignment is commonly understood as “the incorrect timing of a rhythm with respect to another rhythm.”^4^ While this is intuitively correct, a more rigorous definition should account for the manifestations of misalignment, including changes in frequency, amplitude and phase synchrony between at least two oscillatory signals. (B) A typical example is shift work, where overnight wakefulness, eating and physical activity lead to misalignment of melatonin, cortisol and glucose rhythms^3^, ^5^

From a dynamical point of view, disruptions in hormonal rhythms can vary in severity and be either transient (i.e., short‐lived)^16^ or chronic.^17^ In many cases, these disruptions can lead to misaligned hormonal rhythmicity, understood as the loss of either the normal physiological frequency, amplitude or phase synchrony between hormonal rhythms (Figure 1). A discussion on the specific case of circadian misalignment can be found in.^9^ It is also important to distinguish between desynchronization (i.e., loss of phase‐locking) and the broader term of rhythm misalignment. While desynchronization refers exclusively to changes in the oscillatory phase difference between at least two signals, rhythm misalignment implies a deviation in oscillatory properties (not limited to phase) from an accepted standard where rhythms are considered to be ‘aligned’. From an endocrine perspective, aligned hormonal rhythms would correspond to the normal physiological dynamic environment observed in healthy individuals subject to diurnal environmental and behavioural cues (e.g., light–dark cycle, day time physical activity, regular meal timing). This definition immediately calls for additional considerations relating to the intra‐ and interindividual variability in hormonal rhythmicity that can be recognised within a physiological standard. While the statistical quantification of such variability is still the subject of research, mathematical methods can help outline these definitions in nonambiguous, quantitative terms, while at the same time facilitating the implementation of computer algorithms to detect hormonal misalignment and support diagnosis.

## 2 HOW CAN MATHEMATICS INVESTIGATE THE ORIGINS OF HORMONAL MISALIGNMENT?

One of the central questions in the mathematical study of dynamical systems relates to determining the conditions that guarantee the stability of a system's behaviour. This could be used, for example, to determine the kinetic rate values that allow sustained oscillations in biochemical reaction networks.^18^ Mathematical modelling in endocrinology often requires solving these same challenges, with the added complexity that endocrine regulation spans multiple scales of space (e.g., cells, tissues, organs, systems) and time (e.g., from neuronal impulses, secretion and transport phenomena, and gene expression, to daily, monthly and seasonal rhythms).^19^, ^20^ In addition to the question of what regulatory mechanisms lead to oscillatory behaviour, it is also important to determine how oscillatory systems respond to perturbations. While methods for investigating dynamic responses to perturbations and rhythm misalignment are well known in mathematics, they are relatively new in the context of endocrine research. For example, mathematical modelling of the hypothalamic–pituitary–adrenal (HPA) axis has contributed to our understanding of how systems‐level feedback loops underpin ultradian pulsatility of ACTH and glucocorticoid hormones,^21^ how intra‐adrenal feedback loops modulate HPA responses to acute stressors and inflammation,^22^ how dynamic disruptions of the HPA axis can propagate to other endocrine systems,^23^, ^24^ and its cross‐talk interactions with the inflammatory response.^16^, ^25^ While the intensity, duration and frequency of stressors affect the dynamic responses of the HPA axis, hormonal responses are also affected by the timing of perturbations and underlying physiological context. For example, computationally‐derived phase‐response curves have shown that the magnitude of hormonal responses are critically dependent on the timing of the perturbation.^24^, ^26^ Mathematical modelling has also suggested a trade‐off between the HPA axis responses to frequent acute stressors and fitness adaptations allowing anticipation of future conditions, therefore minimising the cost of mismatching new environmental states.^27^

The problem of uncovering the origins of hormonal misalignment is ripe for mathematical and computational modelling. However, suitable dynamic hormone profile data is needed to properly calibrate the models and test their predictions. This means that for circadian misalignment modelling, samples should ideally be collected at a frequency of at least twice per cycle (by the Nyquist theorem), span at least one circadian cycle, and run through protocols that ideally detect multiple analytes. These analytes could range from adrenal and gonadal steroids, catecholamines, peptide hormones (e.g., ACTH, LH/FSH, leptin, insulin), melatonin, to nonhormonal signals such as glucose, metabolites, and inflammatory mediators. Sample collection can also be paired with wearable device technologies that capture the external manifestations of endocrine activity. Time series analysis and machine learning can help determine which wearable signals can constitute reliable clinical correlates of internal hormone rhythmicity and identify computational biomarkers of hormonal misalignment.^14^ This is particularly important to quantify rhythm misalignment in subpopulations stratified by age,^28^ sex,^29^ and other physiological characteristics, including chronotypes^30^ and glucotypes.^31^ The question of how these factors affect the ability of endocrine systems to withstand chronodisruptions is key to develop personalised medicine interventions.^14^

## 3 WHAT MAKES HORMONAL RHYTHMS ROBUST TO SOME PERTURBATIONS BUT FRAGILE TO OTHERS?

Rapid homeostasis of hormonal rhythmicity is usually achieved after minor perturbations such as skipping a meal or going to bed late one night, but misalignment may occur following severe or long‐lasting disruptions such as systemic inflammation or shift work. Since rhythm misalignment often occurs between hormones (and their proxies) regulated by different endocrine axes, we need to investigate when and how endocrine axes interact with each other and whether these cross‐talk interactions facilitate sustained rhythmicity or actively prevent its disruption. This also requires quantifying how much of the observed misalignment originates from the simultaneous perturbation of several endocrine oscillators –perhaps via an upstream effector, and how much is due to the horizontal propagation of the disruption across coupled systems. In other words, we need to understand what mechanisms make endocrine rhythms robust to some perturbations but fragile to others.

Whilst mathematical modelling of endocrine systems is not new,^19^ typically these systems are considered in isolation. Mathematics teaches us that the coupling mechanisms between oscillatory systems may hold the key for guaranteeing their robustness. Therefore, we need to consider not only the organisational principles of endocrine axes but also their cross‐talk interactions across several spatial scales (cellular, tissue, organ, system). By visualising endocrine axes as coupled, nonlinear, pulse‐modulated dynamical systems,^32^ we can also investigate how perturbations in one system propagate to another. Identifying what mechanisms underpin this robustness will also require calibrating mathematical models to experimental data where the impact of perturbations of varying intensity, duration, frequency, timing and context is carefully measured.

**FIGURE 2:**
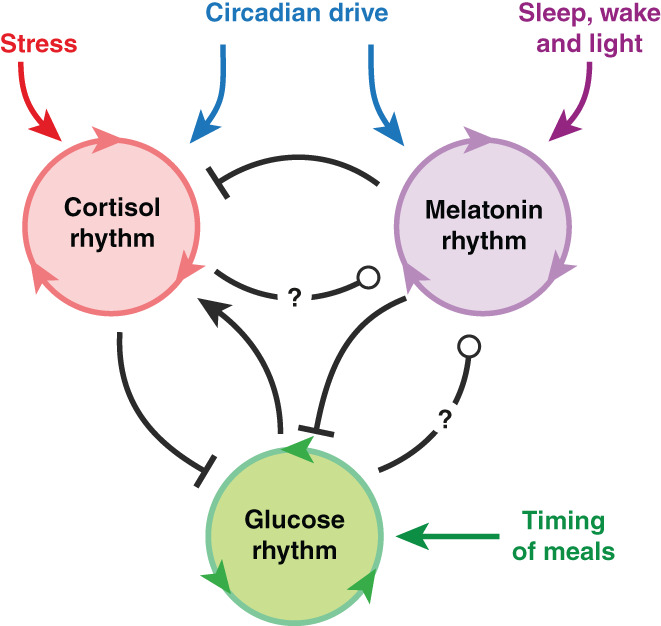
Cross‐talk interactions between oscillatory endocrine systems may facilitate sustained rhythmicity and maintain phase relationships, while also allowing flexible adaptation and entrainment by external cues (e.g., light/dark cycle, timing of meals, stressors). At the same time, coupling mechanisms at different levels of organisation may help ensure robustness to perturbations that could lead to rhythm misalignment

Consider again shift work as an example. Research shows that light does not directly drive melatonin rhythms, but can entrain them. On the other hand, while cortisol rhythms are neither directly driven by ambient light, melatonin can inhibit them.^5^, ^33^ Circadian modulation of glucose metabolism and melatonin dynamics is well known, but only recently the potential antagonism between melatonin and insulin, and the relationship between glucose levels and melatonin secretion, has been explored.^34^ The relationship between the timing of meals and diurnal cortisol dynamics has been documented, including potential adrenal and extra‐adrenal regulatory effects.^35^, ^36^ Thus, a picture emerges where stress, sleep and metabolic rhythms are intertwined, modulated by a circadian hypothalamic master clock, while also subject to modulation by environmental and behavioural cues (Figure 2). Computer simulations can assist in comparing the disruptive effect of different meal timing regimes and of food glycemic index. This may ultimately help design strategies that minimise the misalignment of hormone rhythms while also avoiding overnight hypoglycemia in shift workers.^37^ In this direction, mathematical modelling of the antagonism between the HPA and metabolic axes has explored how normal versus disrupted glucocorticoid dynamics can modulate glucose responses to meals, the circadian variability of such responses, and the modulatory effects of anti‐inflammatory drugs.^23^ Advanced statistical analysis of time series data from continuous hormone measurements can help identify novel features of rhythm misalignment,^38^ complementing existing biomarkers such as cortisol acrophase and dim light melatonin onset (DLMO). Combining these techniques with computer simulations can help postulate the missing coupling mechanisms underpinning misalignment induced by shift work (Figure 2).

## 4 DISCUSSION

A theoretical framework accounting for the coupling between endocrine systems is needed to understand how misalignment of hormonal rhythms and their physical manifestations emerge following perturbations. Computer simulations can support investigations on different endocrine networks (i.e., comparing candidate cross‐talk mechanisms) and their dynamic responses to mistimed cues. This could help probe the origins of rhythm misalignment, identify sources of fragility to external perturbations, as well as their propagation within the endocrine network. Mathematical modelling could be used to understand the difference between rapid versus gradual misalignment in metabolic rhythms,^4^ and determine conditions under which responses to mistimed cues become adaptive or disruptive. This includes mistimed sleep, meals, light exposure and physical activity, all of which are experienced during chronodisruptions such as shift work, jet lag, and systemic inflammation. The goal is to use the insight from these models to predict what perturbations (and under which conditions) would lead to hormonal misalignment and disease.

While mathematical and statistical data analysis techniques can assist in defining concepts such as rhythm misalignment and robustness in quantitative terms, there is also a need to develop theoretical understanding. To achieve this, we need novel quantitative approaches that are appropriately validated against gold standard markers,^15^ including healthy and patient subpopulations stratified by age, sex and physiotypes (e.g., chronotypes and glucotypes). Machine learning techniques can support the identification of novel computational biomarkers of normality, as well as their validation against gold standard markers already in use. However, extracting features from stratified population data –a key step toward personalised diagnosis and therapy– will inevitably require large cohorts. This is because circadian and endocrine regulation are part of a complex, networked system with many interdependencies, with both internal and external factors contributing to it. Therefore, the correct interpretation of computational biomarkers will require considerations about the genetic and phenotypical heterogeneity across populations, as well as the environmental factors contributing to it (e.g., solar time at different latitudes, seasonality, and perhaps even sociocultural pressures). One way to anticipate these developments is to apply mathematical analysis methods to well‐controlled studies in both animal models and humans. These analyses can help piece apart the contributing factors to rhythm misalignment when most variables are controlled, while only allowing to vary those mediating immediate responses to perturbations or those cascading its effects down to other endocrine systems. By themselves, these controlled studies would already constitute a major step forward in disentangling the contribution of each endocrine system to a robust dynamic environment. Ultimately, we need to translate these methodologies into strategies that benefit those individuals most at risk, for example, to mitigate cardiometabolic risk in shift workers.^39^ These calls often invoke the need to consider rhythm adaptations. In this direction, mathematically‐defined concepts such as endocrine flexibility can help characterise the phenotypical changes in endocrine regulation across an individual's lifetime and environmental contexts.^40^

Lastly, despite their potential in healthcare, cheap wearable technologies are not yet used to their full potential, partly because we lack a suitable theoretical framework that explains how the variables they measure relate to the dynamics of hormonal analytes of interest.^14^ As a result, physicians lack the right tools to recognise wearable data as clinical correlates of endocrine dynamics, further delaying their use to monitor health and implement clinical interventions that restore normal rhythmicity. Combining multidimensional datasets with mathematical models will allow the development of algorithms that identify computational biomarkers of rhythm misalignment.^7^ These biomarkers can thus help estimate the health risks associated to rhythm misalignment in personalised ways thanks to the scalability of noninvasive wearable devices.^14^

## AUTHOR CONTRIBUTIONS

**Eder Zavala:** conceptualization; investigation; methodology; writing Original Draft; writing Review Editing.

## CONFLICT OF INTEREST

The author declares that he has no known competing financial interests or personal relationships that could have appeared to influence the work reported in this study.

## Data Availability

Data sharing is not applicable to this article as no new data were created or analyzed in this study.
